# The Method and Experiment of Detecting the Strength of Structural Components Utilizing the Distributed Strain of Sensing Optical Fibers Demodulated by OFDR

**DOI:** 10.3390/s24165212

**Published:** 2024-08-12

**Authors:** Bin Chen, Jun Yang, Dezhi Zhang, Wenxiang Liu, Jin Li, Min Zhang, Ang Li, Zhao Wang

**Affiliations:** National Key Laboratory of Intense Pulsed Radiation Simulation and Effect, Northwest Institute of Nuclear Technology, Xi’an 710024, China; chenbin1@nint.ac.cn (B.C.);

**Keywords:** strength detection, sensing optical fibers, OFDR demodulation technology, stress concentration, distributed strain

## Abstract

Defects occurring during the welding process of metal structural components directly affect their overall strength, which is crucial to the load-bearing capacity and durability of the components. This signifies the importance of accurate measurement and assessment of weld strength. However, traditional non-destructive testing methods such as ultrasonic and non-contact camera inspection have certain technical limitations. In response to these issues, this paper analyzes the detection principle of weld strength, revealing that weld defects reduce the effective area of the structural bearing section and cause stress concentration around them. Through repeated experimental data analysis of samples, strain distribution data along the one-dimensional direction caused by defects such as slag inclusion and porosity were obtained. Experimental results show that this method can identify defect types in welds, including slag inclusion, porosity, and unevenness, and accurately measure the location and size of defects with a precision of 0.64 mm, achieving qualitative analysis of weld defects. Additionally, by deploying distributed optical fiber sensors (DOFS) at different vertical distances along the weld direction, the propagation law of stress induced by different types of weld defects on samples was thoroughly analyzed. This further validates the advantages of this method in weld strength detection, including high spatial resolution, high sensitivity, and non-destructive measurement.

## 1. Introduction

With the rapid development of modern industry, the quality and performance requirements for structural components have become increasingly stringent, especially in fields such as aerospace, automobile manufacturing, and various mechanical containers. The strength and reliability of welded structural components have become critical factors [1,2,3]. In particular, welded joints, as areas prone to welding defects, experience complex and variable stresses. The presence of welding defects poses a serious threat to the safety and stability of the structure [4,5]. During welding, the occurrence of welding defects is relatively common. Effectively reducing the incidence of welding defects and ensuring welding quality meets standard requirements has become a problem that must be addressed in the industrial field [6,7,8]. Welding quality is directly related to the load-bearing capacity and durability of structural components; therefore, accurate measurement and assessment of the strength of welded structural components are particularly important [9,10].

Traditional welding strength measurement methods, such as ultrasonic testing and X-ray inspection, have certain limitations despite their ability to assess welding quality to some extent. For example, these methods often require high operational skills, have slow detection speeds, and cannot achieve continuous real-time monitoring [11,12].

To overcome these limitations, researchers have been exploring more efficient and precise detection technologies. Optical frequency domain reflectometry (OFDR), as a distributed optical fiber sensing technology with high spatial resolution, high sensitivity, and dynamic real-time monitoring capabilities, has demonstrated significant application potential in structural health monitoring [13,14,15]. OFDR technology measures the Rayleigh scattering frequency shift in optical fibers to achieve real-time monitoring of various physical quantities such as temperature and strain, achieving millimeter-level positioning accuracy and a response speed of 100 Hz, making it particularly suitable for precise measurement of strain distributions during the welding process of structural components [16].

Welding defects have many causes, resulting in many kinds of defects. Some defects are on the surface of the weld, and some are in the structure, so it is difficult to find the defects by the naked eye. Most measurement technologies can only detect the surface of the object and the blind area or excessive volume can affect the structural strength of the weld and affect the health monitoring results. Moreover, differing from traditional testing schemes, distributed optical fiber sensors (DOFS) are small in size. Positioning them on weld samples for testing barely affects the structural strength of the welds. Currently, the resolution of DOFS can reach 0.64 mm per point. By placing the sensors adjacent to the welds, real-time health monitoring of the welds can be achieved with virtually no blind spots. Some defects are thinner, with weak structure strength and concentrated strain during stress; some defects are thicker and have more residues, resulting in thick structure strength, less strain, weak edge structure strength and greater strain, so the defect position and defect type can be accurately judged by strain distribution. Optical fibers’ structure is mainly composed of silica, which is not affected by magnetic fields and environmental temperature and will not be easily corroded. It can affect the weld defect monitoring for a long time, find the fatigue damage of the weld position, and the defects in the later stage in time, and reduce unnecessary losses.

By analyzing the detection principle of weld strength, this paper reveals that weld defects reduce the effective area of the structural bearing section and cause stress concentration around them. At this point, using micrometer-sized sensing optical fibers combined with OFDR demodulation technology with micrometer-level spatial resolution to conduct distributed strain measurements around the weld and its surrounding areas can achieve defect detection. This method can distinguish the location of weld defects and defect types such as porosity and slag inclusion, thereby assessing the welding strength of structural components. Through repeated experimental data analysis of samples, strain distribution data along the one-dimensional direction caused by defects such as slag inclusion and porosity were obtained, enabling in-depth analysis of the types and nature of weld defects. Understanding the formation mechanism and influence laws of weld defects also provides strong technical support for improving weld quality and defect repair. Compared with traditional methods, OFDR technology offers higher measurement accuracy, faster response speed, and the ability to achieve continuous real-time monitoring during the welding process, providing new technical means for quality control and performance assessment of welded structural components.

## 2. Measurement Principle and Experimental Setup

### 2.1. Weld Strength Detection Principle Based on OFDR Technology

**Figure 1:**
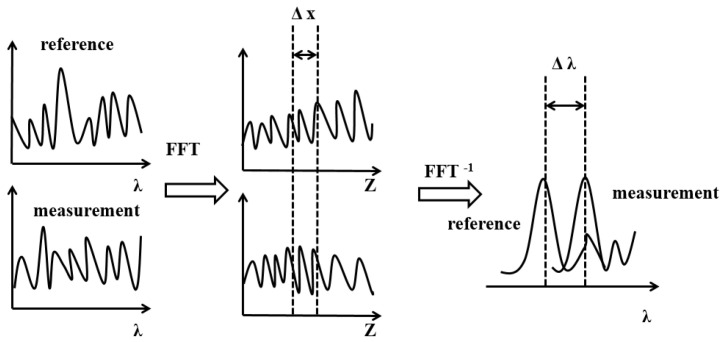
OFDR sensing information demodulation principle [16].

**Figure 2:**
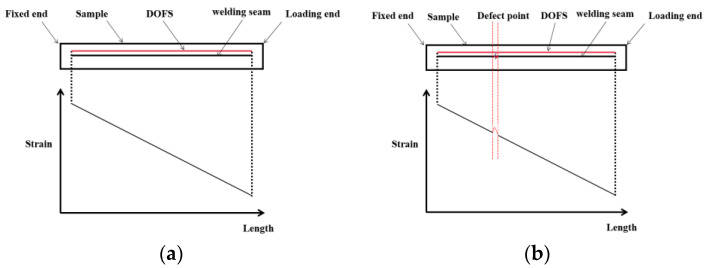
Theoretical strain distribution diagram of welds: (**a**) defect-free sample; (**b**) defective sample.

The OFDR sensing principle is relatively mature. After an optical fiber is produced, its Rayleigh scattering spectrum remains stable. When the environmental temperature or stress applied to the fiber changes, the internal refractive index of the fiber changes, causing a shift in the Rayleigh scattering [17]. The linear relationship between the shift in the Rayleigh backscattering light spectrum at a specific position in the fiber and temperature/strain changes enables high-spatial-resolution distributed optical fiber sensing [18,19,20]. The basic demodulation principle of OFDR is illustrated in Figure 1, where optical frequency domain (time domain) is converted to distance domain (frequency domain) and back to optical frequency domain (to find the spectral shift), thereby demodulating the parameters of the fiber under test. The specific demodulation steps are as follows:

Optical fibers are arranged along the position of the steel plate. The fiber direction is parallel to the weld, the optical fibers are in a close position to the weld joint, and a load is applied at the tail end of the plate. In an ideal state without defects, the strain of the entire steel plate should exhibit a smooth linear curve, as shown in Figure 2a. When there are porosity defects, stress concentration occurs at the defect location, resulting in higher strain than at normal positions, as shown in Figure 2b.

**Figure 3:**
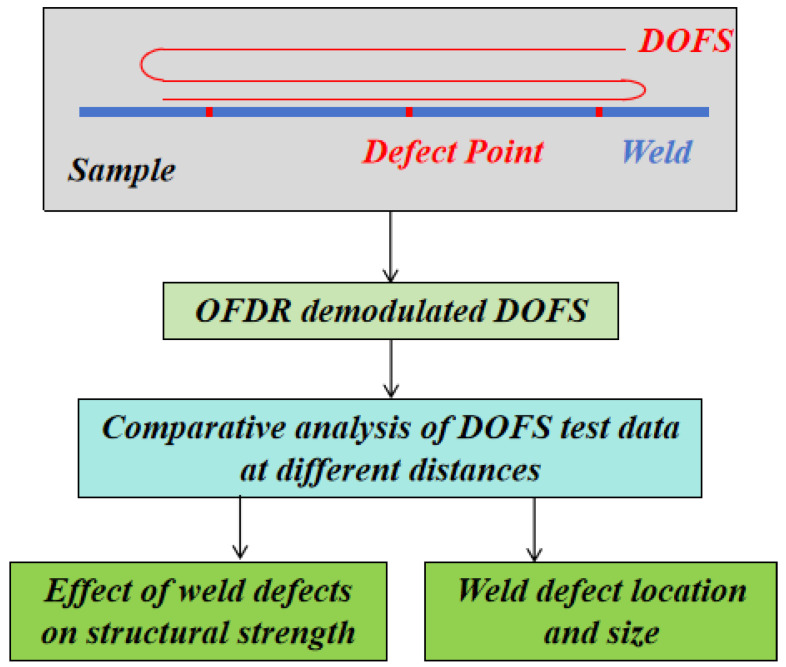
Flowchart of real-time method for detecting weld defects using OFDR distributed optical fiber sensing technology.

**Figure 4:**
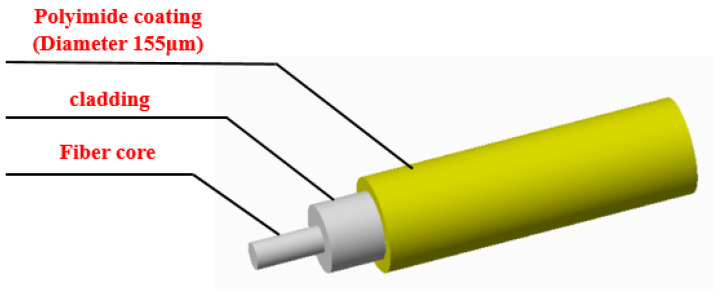
The structure of the sensing fiber used in the experiment.

Figure 3 depicts the flowchart of monitoring weld defects using OFDR distributed optical fiber sensing technology. In this experiment, polyimide-coated optical fiber was used as the sensor. As shown in Figure 4, the diameter of the coating layer was 155 μm, the diameter of the cladding was 125 μm and the working wavelength was 1550 nm~1650 nm. The test sample was securely affixed using 502 instant dry adhesive, and the sample was loaded subsequent to natural curing. The adhesive layer was kept thin, with the combined thickness of the glue and the test fiber amounting to approximately 160 μm. Consequently, the influence of the glue on the strain test results was deemed negligible. The strain results after applying a load to the samples were tested using OFDR distributed optical fiber sensing equipment. The test data from the two samples were compared and analyzed to determine the location and size of weld defects and compare the impact of the two types of weld defects on the structure.

### 2.2. Experimental Setup

**Figure 5:**
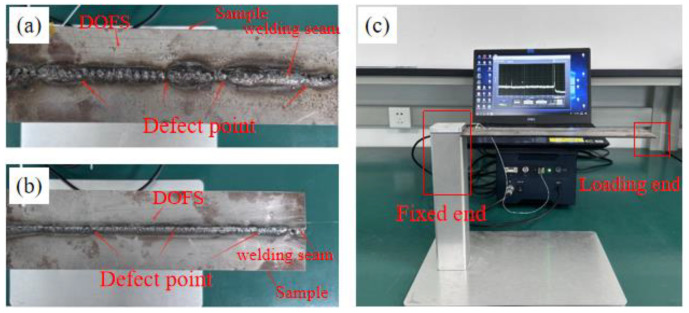
(**a**) Slag inclusion defect sample; (**b**) porosity defect sample; (**c**) experimental platform setup.

Experimental samples with porosity and slag inclusion defects were prepared using different welding processes. Figure 5 shows the distributed sensor with a polyimide-coated optical fiber. The test specimen employed a 3 mm thick steel plate, fashioned into a rectangular shape with dimensions of 350 mm in length and 80 mm in width. At the center of this rectangular plate, a section measuring 50 mm in length and 21 mm in width was designated as the test area, which was integrated with a fixed plate. Subsequently, the entire middle portion of the plate was divided into two halves along its longitudinal axis, and the resulting cut surfaces of the steel plate were then welded back together, adhering to a predetermined cutting pattern. This precise welding position on the cut surface ensured a seamless reunion. The optical fibers were subsequently laid either directly along the weld seam or parallel to the cutting direction, ensuring that their orientation remained parallel to the weld joint. To further analyze the impact of varying distances from the weld, several polyimide-coated fibers were strategically positioned at different intervals from the weld. Specifically, as shown in Figure 5a, five different distances of DOFS were arranged along the weld axis of the slag inclusion defect sample, and four different distances were arranged for the porosity defect sample, as shown in Figure 5b. After sample preparation, as shown in Figure 5c, a weight block was used to apply a load to the tail end of the sample. The far right end of Figure 5a,b is the loading end and the strain distribution at various positions during loading was demodulated in real time using an OSI-S demodulation device.

## 3. Results Analysis and Discussion

### 3.1. Weld Strength Analysis with Slag Inclusion Defects

**Figure 6:**
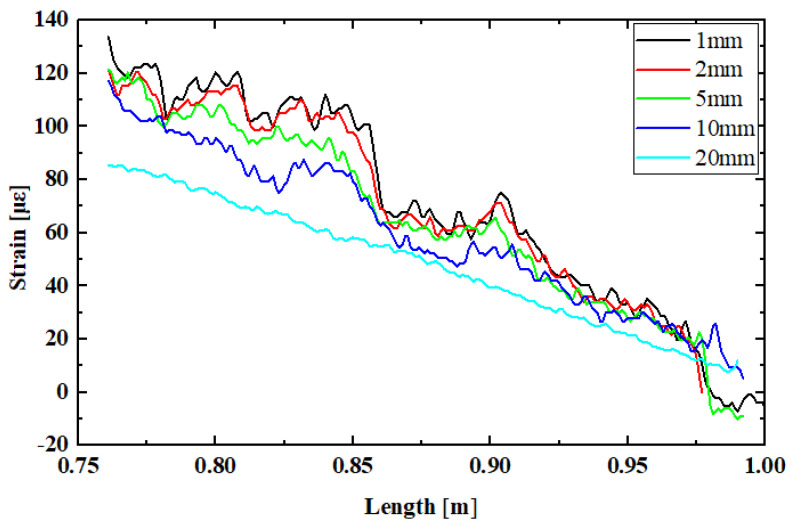
Distributed strain curves of fiber sensors at different deployment distances along the weld direction with slag inclusion defect characteristics.

The slag inclusion defect sample in Figure 5a was arranged with sensing optical fibers at distances of 1 mm/2 mm/5 mm/10 mm/20 mm from the weld, according to the deployment method in Figure 2. By reading the strain data from the OFDR demodulator, distributed strain curves for fiber sensors at different deployment distances along the weld direction with slag inclusion defect characteristics were obtained, as shown in Figure 6. Using the high-spatial-resolution strain data from OFDR, it is intuitively observable that the overall strain decreased as the distance from the weld increased, indicating that the structural strength at the weld was lower than that of the steel plate itself. During loading, the area with reduced strength due to the weld affected the sample up to a distance of approximately 20 mm from the weld. Analyzing the distributed strain data at 1 mm showed that the slag inclusion distribution in this sample was large, significantly impacting the weld strength.

**Figure 7:**
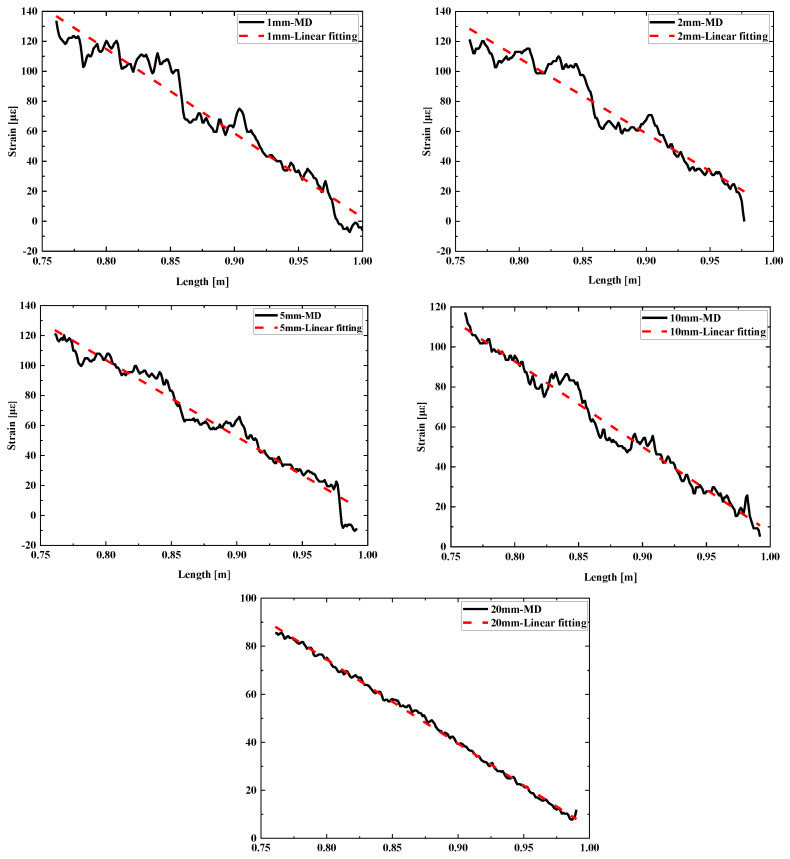
Comparison of measurement curves and fitting curves for welds with slag inclusion defects.

The measurement data from fibers deployed at five different distances were fitted, as shown in Figure 7.

Upon subjecting the weld sample to loading at its tail, we observed a strain distribution that resembled that of a beam structure, featuring a linear progression. Specifically, the strain at the loading end was minimal, gradually increasing towards the fixed end where it attained its maximum value. We hypothesize that for a flawless strain distribution, a linear fit would aptly describe the observed strain profile. When the measured strain exceeds the fitted linear value, it suggests the presence of a thin structural region prone to stress concentration and heightened strain levels, thereby rendering it more susceptible to damage. Conversely, if the measured strain is lower than the fitted value, it may indicate the existence of residual material or a thicker structural section, both of which would result in reduced strain magnitudes.

It can be seen that the strain curves tested by fibers at distances of 1 mm to 10 mm from the weld exhibited fluctuations compared to the fitting results, primarily manifested as larger or smaller strain data at multiple locations. This can be explained by the presence of residual slag of different sizes at each position within the weld, while the smaller strain data occurred at the sharp peaks of slag inclusion edges, where the effective area of the structural bearing section was smaller, leading to more concentrated stress and easier cracking. As the distance from the weld increased, the overall strain decreased, and the influence of the weld on the structure became smaller. However, the defect locations detected at different distances were basically consistent. At a distance of 20 mm from the weld, the strain was basically linear, with no obvious larger or smaller strain values, indicating that the fiber could no longer detect the weld defect location, further confirming that closer distances yielded higher detection accuracy.

### 3.2. Weld Strength Data Analysis with Porosity Defects

**Figure 8:**
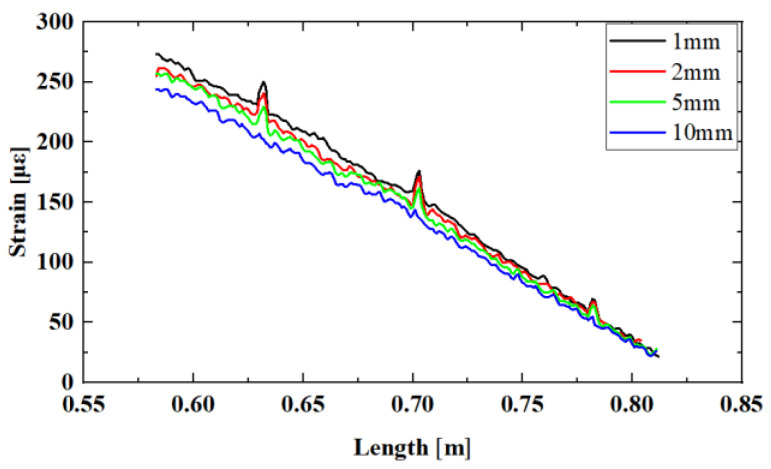
Distributed strain curves of fiber sensors at different deployment distances along the weld direction with porosity defect characteristics.

To verify the influence of defect characteristics on the component strength measurement method based on distributed strain, sensing optical fibers were arranged at distances of 1 mm/2 mm/5 mm/10 mm from the weld for the porosity defect sample in Figure 5b, following the deployment method in Figure 2. As shown in Figure 8, strain measurement data for four one-dimensional distributions along the weld direction with porosity defects were obtained. Unlike the strain distribution pattern caused by slag inclusion defects, stress concentration caused by porosity characteristics was more evident, aiding in precise defect location. It is clearly observable that this sample had three significant porosity defects. This indicates that, at smaller-sized defect points, porosity had a relatively small impact on the overall strength of the structural component but responded rapidly to the strength of the weld itself. This characteristic is highly beneficial for the OFDR distributed strain measurement method.

**Figure 9:**
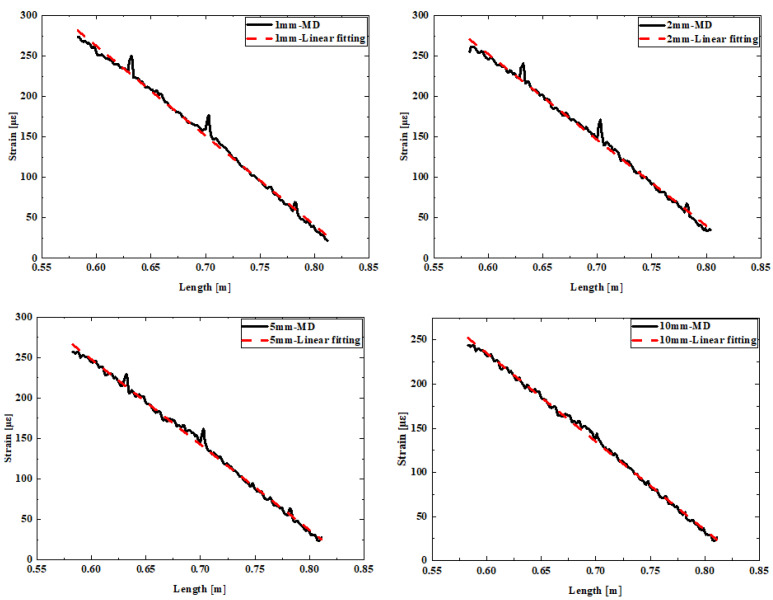
Comparison between measurement curve and fitting curve of the weld with slag inclusion defect.

To further validate the above conclusions, linear fitting was performed on the fiber curves at four positions, as shown in Figure 9. Due to the smaller effective area of the bearing section at the porosity defect location, stress was more concentrated. It can be observed from the figure that the fiber test data at distances of 1 mm to 5 mm from the weld all showed three locations with larger strain values. By reading the peak positions of the waveforms, the accurate locations of the porosity defects were obtained, which were 0.625 m, 0.700 m, and 0.780 m, respectively. From the distributed strain curves, the strength influence caused by stress concentration due to porosity defects was relatively small for the overall sample. At 10 mm, the test data were basically linear, and the stress concentration phenomenon caused by porosity almost disappeared, indicating that the fiber could no longer detect the influence of porosity defects on the structure at this point. This further confirms that porosity defects had a weaker impact on the overall strength of the sample compared to slag inclusion defects.

## 4. Conclusions

In this experimental study, OFDR distributed optical fiber sensor technology was used to monitor the strain distribution along the entire weld. Through OFDR test results, the strain distribution along the entire weld could be clearly observed, enabling accurate judgment of weld quality and precise location of defective weld positions. It is noteworthy that weld defects of different sizes had significant differences in their impact range on the structure. OFDR distribution data showed that slag inclusion defect locations may exhibit smaller or larger strain changes, while porosity defect locations only exhibited larger strain changes. This indicates that OFDR distributed optical fiber sensing technology can be used to monitor weld defect locations and sizes and analyze weld defect types through test results. Irrespective of their position, whether residing on the surface or embedded within the interior of the weld, defects had a pronounced impact on the structural integrity and strength of the component. This influence manifested in the form of distinctive strain patterns observed during loading, differing notably from those exhibited by components featuring flawless weld structures. Consequently, the employment of DOFS-based OFDR technology presents a robust solution for detecting and continuously monitoring defects, both those situated at the surface and those buried deep within the weld.

OFDR technology for weld quality inspection not only possesses high precision and sensitivity but also enables real-time monitoring and remote control. The optical fiber boasts a minuscule volume, employing an instantaneous dry adhesive as its fixed mode. This resulted in a remarkably thin overall construction that did not alter the weld structure, thereby ensuring a negligible impact on the entirety of the test data from the sensor. This not only provides a comprehensive understanding of the formation mechanisms and influence laws of weld defects but also offers strong technical support for improving weld quality and defect repair. Through extensive experimental data analysis, this paper provides effective insights for various structural component strength monitoring and analysis.

## Data Availability

The data presented in this study are available on request from the corresponding author. The data are not publicly available due to privacy concerns.
